# A theory of attentional modulations of the supratemporal generation of the auditory mismatch negativity (MMN)

**DOI:** 10.3389/fnhum.2014.01065

**Published:** 2015-01-29

**Authors:** Tom A. Campbell

**Affiliations:** Neuroscience Center, University of HelsinkiHelsinki, Finland

**Keywords:** auditory mismatch negativity (MMN), early selective attention effect, selective attention, supratemporal cortex, auditory Event-Related Potentials (ERP), corticofugal connections, olivocochlear system, early filter

“*Everyone knows what attention is*. It is the taking possession by the mind in clear and vivid form, of one out of what seem several simultaneously possible objects or trains of thought. Focalization, concentration, of consciousness are of its essence. It implies withdrawal from some things in order to deal effectively with others, and is a condition which has a real opposite in the confused, dazed, scatterbrained state which in French is called distraction, and Zerstreutheit in German,” thus wrote James (1890, pp. 403–404). In the field of auditory cognitive neuroscience, it would seem everyone knows what attention is not—the generation of the auditory mismatch negativity (MMN).

However, Erlbeck et al.'s (2014) results show that attention affects the auditory MMN component that can contribute to the auditory Long-Latency Responses (ALLRs) of the auditory Event-Related Potential (ERP); an ERP component generated by the electrochemical responses of populations of neuronal elements firing synchronously in response to unexpected acoustical changes in sequences of auditory stimuli. Erlbeck et al.'s findings contest the received wisdom that this auditory MMN component is elicited in a task-independent unconscious pre-attentive manner. Erlbeck et al.'s interpretation is best-placed with respect to the history of auditory cognitive neuroscience, to which this commentary turns, leading into an appraisal of the implications of Erlbeck et al. study for understanding MMN and auditory attention.

Hillyard et al. (1973) originally identified the “early selective attention effect” as a scalp negativity occurring during the first major negative deflection of the ALLRs of the auditory ERP: the N1. An oddball sequence—comprised of a repeated standard *S* tone, interspersed amongst which was an occasional unexpected deviant tone *D* differing in terms of one attribute (pitch) of the form *SSSSSSSSDSSSSD*—was presented to either ear, that is, a different oddball sequence to each ear. Sounds were binaurally asynchronous and the participant counted only pitch-deviant tones presented to one attended ear. The early selective attention effect revealed was an enhanced N1 deflection elicited by a sound presented to the attended rather than the to-be-ignored ear.

Näätänen et al. (1978) demonstrated attention to standards elicited a long-lasting processing negativity, thought to contribute to the componentry of phenomena kindred to this early selective attention effect. Näätänen et al. also revealed that pitch deviants yet not standards elicited a long-lasting negativity at sites over the temporal lobes—the auditory MMN that occurs in response to any deviant. This MMN was followed by an intensified P3 deflection in response to just the attended deviants. Accordingly, Näätänen et al. isolated the auditory MMN from the processing negativity (PN) reflecting selective attention via early filter processes or an “attentional set.” Auditory MMN was similarly isolated from the mechanisms of late attentional selection or “response set” manifest in the generation of an increased P3 by the attended deviant yet neither the attended standards nor the to-be-ignored sounds. The increased P3 deflection was arguable caused by elicitation of the P3b component (Sutton et al., 1965; Kok, 2001; Polich, 2003).

Investigations since have demonstrated an influence of attention on MMN (Woldorff et al., 1991; Woods et al., 1992; Oades and Dittmann-Balcar, 1995; Dittmann-Balcar et al., 1999). Tenably, some of these attentional effects have rather reflected an overlapping N2b component (Van Zuijen, 2006). Yet in Erlbeck et al.'s “ignore” task, during which the participant's attention is drawn away from the to-be-ignored auditory stimulation, rather than just ignoring that sound, the study revealed that the mastoid-indexed supratemporal generation of the MMN componentry is influenced by attention—a polarity reversal at the mastoids (Figure 1) unprecedented for the N2b.

**Figure 1:**
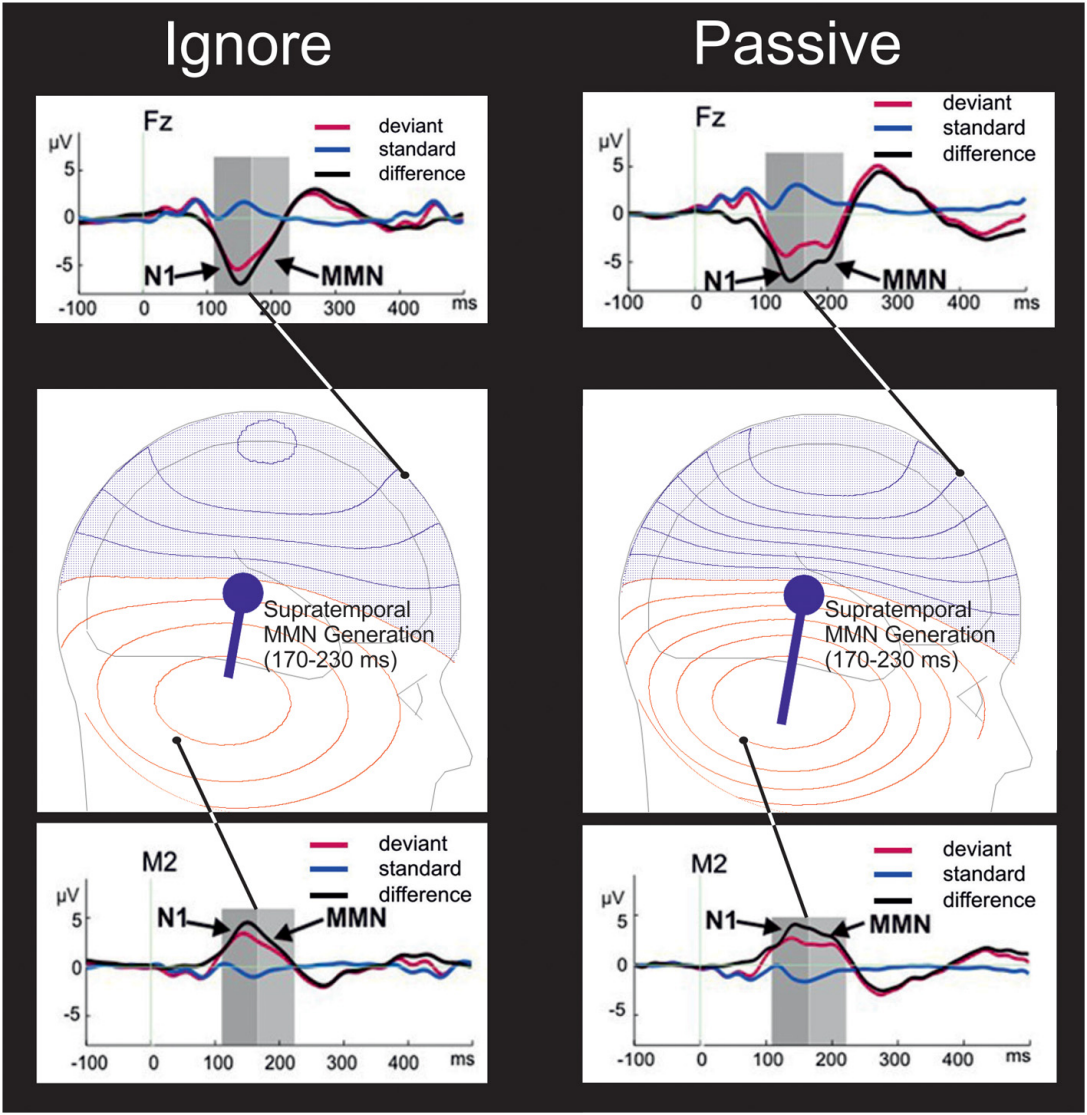
**Theoretical schematic of the influence of attention on supratemporal MMN generation**. In the “ignore” condition **(left panels)**, participants performed a visual task in which attention is drawn away from the to-be-ignored auditory stimulation by a primary visual task of detecting a scene in a silent movie. In the “passive” task **(right panels)** participants watched that movie whilst just ignoring the sound. As illustrated in the middle row of panels, attention to the sounds in the passive task is assumed to increase supratemporal MMN generation by a population of tens of thousands of similarly oriented axons/dendrites of neuronal elements firing simultaneously within each hemisphere in response to auditory deviance, the cumulative action of which can be approximated by a dipolar source of a primary current (denoted by a blue circle) in each hemisphere giving rise to volume currents passing through the brain, liquor, skull, and scalp to Electroencephalogram (EEG) electrodes. The moment of that dipole – represented by the length of a straight line vector originating from the dipolar source location tangentially oriented to the skull – is reduced by the engagement of visual attention during the “ignore” task, attenuating the amplitude of the negative voltage (blue isopotential contours) of the frontally maximal MMN (upper row of panels) and also attenuating the corresponding amplitude of the polarity reversal; a positive voltage (red isopotential contours) at the mastoids (lower row of panels). Each neighboring contour describes a fixed step in scalp potential. Thus, as apparent at the electrodes shown (black dots), with a higher density of such isopotential contours simulated in the passive condition, both MMN at a frontal electrode (Fz) and the MMN polarity reversal at the right mastoid (M2) exhibited higher amplitudes in the passive than in the ignore task. As depicted by the observed deviant-standard difference waves in the upper and lower rows of panels, there is an increase of the N1 deflection (Jacobsen and Schröger, 2003) maximal in amplitude 110–170 ms post-onset onto which is superimposed the second maximal negativity (right panel), the observed MMN. This duration-decrement MMN was qualitatively less apparent in the “ignore” task (left panel) due to a reduction in supratemporal MMN generation within the time range 170–230 ms post-stimulus. It is worth noting that the reference problem might have been a concern: the ERP depicted at M2 was recorded against a common-average reference, whilst the ERPs at Fz were recorded against an averaged-mastoids reference. Accordingly, the mastoidal MMN enhancement illustrated might have carried contributions from effects apparent at frontocentral channels with an averaged-mastoids reference—channels including the electrode Fz for which ERPs are depicted. Such frontocentral contributions might have encompassed that of N2b generation, which is arguably superimposed over a long-lasting MMN plotted for the Fz electrode over the frontal scalp. However, a re-referencing (Supplemental Data Figure A) stood as tentative counter-evidence against any strong view that the depicted attentional modulation of MMN at the mastoid was rather a pure N2b. This new re-referencing employed a reference electrode non-ideally located on the lower canthus of the right eye (IO2), though advantageously situated below the Sylvian fissure. This re-referencing revealed in the grand-averaged difference waves at the M2 electrode a near-identical pattern of average MMN polarity reversals with respect to attentional condition. These polarity reversals peaked around 160 ms and went relatively uninfluenced by supratemporal N1 and N2b generation. Using BESA (Berg and Scherg, 1994) Simulator 1.0, isopotential contours were projected onto the depictions of the scalp from theoretically corroborative bilateral pairs of supratemporal MMN dipoles with only their moments being assumed to be influenced by attention. Adapted with permission from Erlbeck et al. (2014).

As Erlbeck et al. recorded potentials at each scalp electrode relative to a mastoid electrode, to assess this intriguing attentional modulation of this polarity reversal, necessitated an oft-used approach (common-average re-referencing) of subtracting the average of the signals at all electrodes from the signal at each electrode including the mastoid electrodes of interest. It is worth noting that the benchmark system of electrodes used, the 10–20 system, more densely samples the upper than the lower half of the scalp; a spatial sampling from which such a common-average re-referencing (Virtanen et al., 1996, 1998) could, at least in principle, cause an attentional modulation of the observed frontocentral negativity broadly distributed over the upper scalp to migrate from frontocentral electrodes becoming a polarity reversal of that negativity at the mastoids. Considering this concern, a previous investigation, albeit within a distinct paradigm, reassuringly showed that auditory ERPs derived from electroencephalogram (EEG) recorded relative to an electrode on the tip-of-the-nose exhibited attentional influences on the amplitude of polarity reversal of auditory MMN at the mastoids apparent in the grand-averaged ERPs (Sussman et al., 2003). Further corroboration stems from attentional modulation of supratemporally generated MMNm responses—the magnetically measured counterpart of this polarity reversal—recorded in a “referenceless” manner with respect to no particular location on the scalp using magnetoencephalographic techniques (Woldorff et al., 1998). The weight of evidence steers the interpretation of Erlbeck et al.'s polarity reversal away from methodological nuances of the EEG analysis and ERP derivation, more toward a *bona fide* attentional modulation of supratemporal MMN.

A kindred phenomenon to this attentional modulation of supratemporal MMN is the sensory-specific N1 Component 2 of Näätänen and Picton (1987)—comprising the N1a, Tc, and N1c(Tb) maximal at temporal sites (Woods, 1995)—known to be modulated by attention, in that there is an attenuation of this N1c component that is seen when people are instructed to ignore rather than attend-to the sounds; be that attention passive (Perrault and Picton, 1984) or be that attention active (Snyder et al., 2006). However, the radial generation of the attentional N1c enhancement and the somewhat earlier timing (120–170 ms post-stimulus onset, Woods, 1995) renders it difficult to imagine how that N1c might strongly contribute to the attentional increase of supratemporal MMN revealed by Erlbeck et al.

Postulated in a tenable model (Sussman, 2007), subsequently revised (Sussman et al., 2014), is that attentional task effects and top-down control can centrally modulate sound organization and standard formation process comprising the stimulus-driven formation of auditory objects and their seriation into auditory streams; attentional task effects, in turn, indirectly influencing the dependent processes of deviance detection that occur when a deviant is presented. Yet a question-mark resides in that model over whether there are comparable direct influences of attention on acoustical deviance detection.

As visible in the insets of Figure 1, considering the comparison of ERPs in response to the standards, there was an attentional influence upon the frontocentrally distributed P2 deflection, a visible tendency exhibiting a polarity reversal less apparent at the mastoid electrode M2. At first blush, were this frontocentral P2 enhancement (ignore < passive) significant, such an attentional influence might support this model's notion of attentional influences on neurocognitive processes of sound organization and standard formation. Harris et al. (2012) reveal an identifiable attentional P2 amplitude advantage (passive < focused) in auditory ERPs to standard sounds. Yet any such significant support for the model from P2 amplitudes would be partial in that Erlbeck et al. reveal the converse effect to Harris et al. such that at frontocentral electrodes, the pattern of mean P2 amplitudes in ERPs to standards is focused < ignore < passive.

In a sense, Sussman et al.'s model sophisticatedly reconciles attentional influences upon MMN generation with the disparate notion that MMN goes unmodulated by attention: MMN is modulated by auditory scene analysis, by how the auditory information is structured in memory, not by attention itself. This paradox holds explanatory power, yet as such remains an impasse. Parsimony exists in the question-mark assumption of Sussman et al.'s model, the supposition of direct attentional influences on the detection of acoustical deviance—a conjecture, once made, raising new questions.

In assessment, after Erlbeck et al.'s contribution, the question—now assuming a direct attentional influence on the MMN generation, an attentional influence that supports deviance detection—can no longer be if supratemporal MMN generation is affected by attention but rather what form of attention could be affecting supratemporal MMN? When a cat visually attends a mouse, subcortical auditory responses of the dorsal cochlear nucleus are reduced (Hernández-Peon et al., 1956). Attention to a visual discrimination task reduces responses of the auditory nerve to clicks (Oatman, 1971; Oatman and Anderson, 1977). In humans, attention to the visual modality also reduces auditory nerve responses (Lukas, 1980) and otoacoustic emissions evoked by a click (Puel et al., 1988).

A viable theoretical interpretation is that these influences of visual attention on subcortical mechanisms are directly mediated through a feedback system including the supratemporal cortex by some of the many top-down routes of the descending auditory system via corticofugal connections and the olivocochlear system, in turn affecting the bottom-up flow of information from auditory stimulation upward through the ascending auditory system. Corticofugal projections (Ayala and Malmierca, 2013) descending from supratemporal cortex, particularly regions of supratemporal cortex influenced by deviance, accordingly act as attentional filters that enhance task-relevant (deviant) stimuli and reduce task-irrelevant (deviant) stimuli (Nuñez and Malmierca, 2007). That is, when Erlbeck et al.'s “ignore” task is to attend to visual stimulation and ignore irrelevant auditory stimuli, the task demands of visual attention leads to this top-down corticofugal filtering and, in turn, an attenuated processing of task-irrelevant auditory deviance within supratemporal cortex.

A prediction derived from this theory is that using Erlbeck et al.'s new paradigm, which investigates the effects of drawing attention away from auditory stimulation with a visual task, is that the mastoid polarity reversal of the MMN shown at frontocentral sites would be attentionally modulated, as shown in Erlbeck et al.'s paper, but also when EEG at the mastoids is recorded relative to an electrode location that is unlikely to pick up frontocentral contributions, such as the tip-of-the-nose. Additional theoretical predictions are that responses derived from human invasive intracranial recordings, as well as source-reconstructed magnetoencephalogram (MEG) and EEG, would exhibit a modulation of supratemporal MMN generation in this new paradigm. MEG source localization of the generation of auditory brainstem responses to clicks (Parkkonen et al., 2009) is now, in principle, extendible to subcortical responses to complex sounds (Skoe and Kraus, 2010; Campbell et al., 2012). Reconstruction of the MEG sources active during auditory investigations could in turn feasibly assess the existence and nature of the attentional modulations of the postulated functional interactions between sub-cortical and cortical sources. At such a source level, the reference problem is not a problem.

## Conflict of interest statement

The author declares that the research was conducted in the absence of any commercial or financial relationships that could be construed as a potential conflict of interest.

## References

[B1] Ayala Y. A. Malmierca M. S. (2013). Stimulus-specific adaptation and deviance detection in the inferior colliculus. Front. Neural Circuits 6:89. 10.3389/fncir.2012.00089 23335883 PMC3547232

[B35] Berg P. Scherg M. (1994). A fast method for forward computation of multiple-shell spherical head models. Electroencephalogr. Clin. Neurophysiol. 90, 58–64. 10.1016/0013-4694(94)90113-9 7509274

[B2] Campbell T. Kerlin J. R. Bishop C. W. Miller L. M. (2012). Methods to eliminate stimulus transduction artifact from insert earphones during electroencephalography. Ear Hear. 33, 144–150. 10.1097/AUD.0b013e3182280353 21760513 PMC3214253

[B3] Dittmann-Balcar A. Thienel R. Schall U. (1999). Attention-dependent allocation of auditory processing resources as measured by mismatch negativity. Neuroreport 10, 3749–3753. 10.1097/00001756-199912160-00005 10716203

[B4] Erlbeck H. Kübler A. Kotchoubey B. Veser S. (2014). Task instructions modulate the attentional mode affecting the auditory MMN and the semantic N400. Front. Neurosci. 8:654. 10.3389/fnhum.2014.00654 25221494 PMC4145469

[B5] Harris K. C. Wilson S. Eckert M. A. Dubno J. R. (2012). Human evoked cortical activity to silent gaps in noise: effects of age, attention, and cortical processing speed. Ear Hear. 33, 330–339. 10.1097/AUD.0b013e31823fb585 22374321 PMC3340542

[B6] Hernández-Peon R. Scherrer H. Jouvet M. (1956). Modification of electric activity in cochlear nucleus during attention in unanesthetized cats. Science 123, 331–332. 10.1126/science.123.3191.331 13298689

[B7] Hillyard S. A. Hink R. F. Schwent V. L. Picton T. W. (1973). Electrical signs of selective attention in the human brain. Science 182, 177–180. 10.1126/science.182.4108.177 4730062

[B8] Jacobsen T. Schröger E. (2003). Measuring duration mismatch negativity. Clin. Neurophysiol. 114, 1133–1143. 10.1016/S1388-2457(03)00043-9 12804682

[B9] James W. (1890). The Principles of Psychology. New York, NY: H. Holt and Company 10.1037/11059-000

[B10] Kok A. (2001). On the utility of P3 amplitude as a measure of processing capacity. Psychophysiology 38, 557–577. 10.1017/S0048577201990559 11352145

[B11] Lukas J. H. (1980). Human auditory attention: the olivocochlear bundle may function as a peripheral filter. Psychophysiology 17, 444–452. 10.1111/j.1469-8986.1980.tb00181.x 7465714

[B12] Näätänen R. Gaillard A. W. Mäntysalo S. (1978). Early selective-attention effect on evoked potential reinterpreted. Acta Psychol. (Amst.) 42, 313–329. 10.1016/0001-6918(78)90006-9 685709

[B13] Näätänen R. Picton T. (1987). The N1 wave of the human electric and magnetic response to sound: a review and an analysis of the component structure. Psychophysiology 24, 375–425. 10.1111/j.1469-8986.1987.tb00311.x 3615753

[B14] Nuñez A. Malmierca E. (2007). Corticofugal modulation of sensory information. Adv. Anat. Embryol. Cell Biol. 187, 1–74. 10.1007/978-3-540-36771-0 17212068

[B15] Oades R. D. Dittmann-Balcar A. (1995). Mismatch negativity (MMN) is altered by directing attention. Neuroreport 6, 1187–1190. 10.1097/00001756-199505300-00028 7662904

[B16] Oatman L. C. (1971). Role of visual attention on auditory evoked potentials in unanesthetized cats. Exp. Neurol. 32, 341–356. 10.1016/0014-4886(71)90003-3 5110221

[B17] Oatman L. C. Anderson B. W. (1977). Effects of visual attention on tone burst evoked auditory potentials. Exp. Neurol. 57, 200–211. 10.1016/0014-4886(77)90057-7 891689

[B18] Parkkonen L. Fujiki N. Mäkelä J. P. (2009). Sources of auditory brainstem responses revisited: contribution by magnetoencephalography. Hum. Brain Mapp. 30, 1772–1782. 10.1002/hbm.20788 19378273 PMC6870971

[B19] Perrault N. Picton T. W. (1984). Event-related potentials recorded from the scalp and nasopharynx. I. N1 and P2. Electroencephalogr. Clin. Neurophysiol. 59, 177–194. 10.1016/0168-5597(84)90058-3 6203709

[B20] Polich J. (2003). Theoretical overview of P3a and P3b, in Detection of Change: Event-Related Potential and fMRI Findings, ed Polich J. (Norwell, MA: Kluwer Academic Press), 83–98.

[B21] Puel J. L. Bonfils P. Pujol R. (1988). Selective attention modifies the active micromechanical properties of the cochlea. Brain Res. 447, 380–383. 10.1016/0006-8993(88)91144-4 3390709

[B22] Skoe E. Kraus N. (2010). Auditory brainstem response to complex sounds: a tutorial. Ear Hear. 31, 302–324. 10.1097/AUD.0b013e3181cdb272 20084007 PMC2868335

[B23] Snyder J. S. Alain C. Picton T. W. (2006). Effects of attention on neuroelectric correlates of auditory stream segregation. J. Cogn. Neurosci. 18, 1–13. 10.1162/089892906775250021 16417678

[B24] Sussman E. (2007). A new view on the MMN and attention debate: auditory context effects. J. Psychophysiol. 21, 164–175 10.1027/0269-8803.21.34.164

[B25] Sussman E. S. Chen S. Sussman-Fort J. Dinces E. (2014). The five myths of MMN: redefining how to use MMN in basic and clinical research. Brain Topogr. 27, 553–564. 10.1007/s10548-013-0326-6 24158725 PMC4000291

[B26] Sussman E. Winkler I. Wang W. (2003). MMN and attention: competition for deviance detection. Psychophysiology 40, 430–435. 10.1111/1469-8986.00045 12946116

[B27] Sutton S. Braren M. Zubin J. John E. R. (1965). Evoked-potential correlates of stimulus uncertainty. Science 150, 1187–1188. 10.1126/science.150.3700.1187 5852977

[B28] Van Zuijen T. (2006). Sensory Auditory Processing and Intuitive Sound Detection: An Investigation of Musical Experts and Nonexperts. F.T. dissertation, University of Helsinki, Finland. Available online at: http://ethesis.helsinki.fi/julkaisut/kay/psyko/vk/vanzuijen/.

[B29] Virtanen J. Ahveninen J. Ilmoniemi R. J. Näätänen R. Pekkonen E. (1998). Replicability of MEG and EEG measures of the auditory N1/N1m-response. Electroencephalogr. Clin. Neurophysiol. 108, 291–298. 10.1016/S0168-5597(98)00006-9 9607518

[B30] Virtanen J. Rinne T. Ilmoniemi R. J. Näätänen R. (1996). MEG-compatible multichannel EEG electrode array. Electroencephalogr. Clin. Neurophysiol. 99, 568–570. 10.1016/S0013-4694(96)96575-X 9020817

[B31] Woldorff M. G. Hackley S. A. Hillyard S. A. (1991). The effects of channel-selective attention on the mismatch negativity wave elicited by deviant tones. Psychophysiology 28, 30–42. 10.1111/j.1469-8986.1991.tb03384.x 1886962

[B32] Woldorff M. G. Hillyard S. A. Gallen C. C. Hampson S. R. Bloom F. E. (1998). Magnetoencephalographic recordings demonstrate attentional modulation of mismatch-related neural activity in human auditory cortex. Psychophysiology 35, 283–292. 10.1017/S0048577298961601 9564748

[B33] Woods D. L. (1995). The component structure of the N1 wave of the human auditory evoked potential. Electroencephalogr. Clin. Neurophysiol. Suppl. 44, 102–109. 7649012

[B34] Woods D. L. Alho K. Algazi A. (1992). Intermodal selective attention. I. Effects on event-related potentials to lateralized auditory and visual stimuli. Electroencephalogr. Clin. Neurophysiol. 82, 341–355. 10.1016/0013-4694(92)90004-2 1374703

