# Supplementary material for: A theory of attentional modulations of the supratemporal generation of the auditory mismatch negativity (MMN)
Source: Front Hum Neurosci. 2015 Jan 29;8:1065. doi: 10.3389/fnhum.2014.01065 (PMC4310267; doi:10.3389/fnhum.2014.01065)
Supplement: Supplementary file 1 [file Image1.PDF]

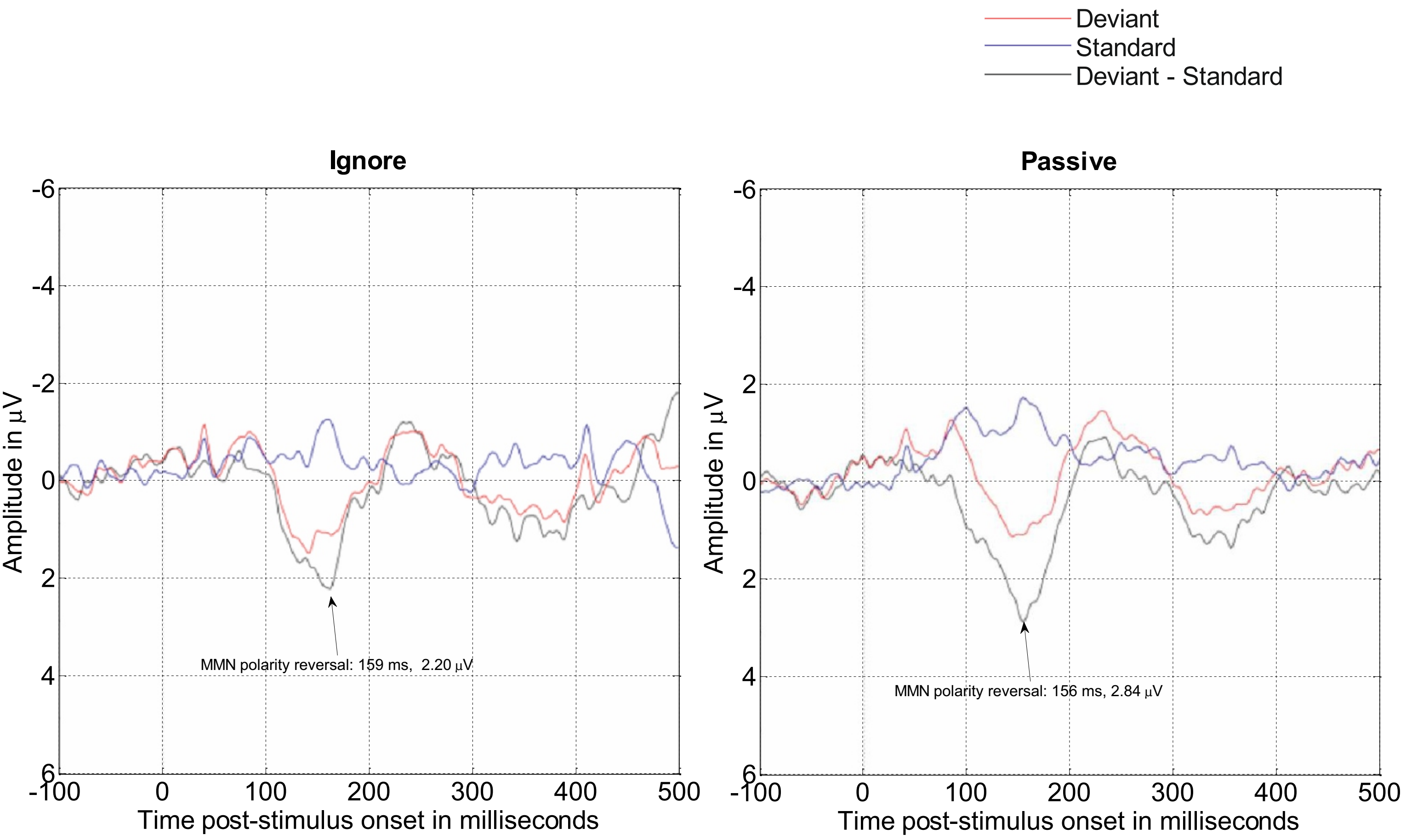

Figure A: IO2-referenced M2 Grand-averaged ERPs as a function of auditory stimulus deviance and the corresponding difference wave as for the "ignore" and "passive" conditions of Erlbeck *et al.*
